# Therapeutic targeting of replicative immortality

**DOI:** 10.1016/j.semcancer.2015.03.007

**Published:** 2015-12

**Authors:** Paul Yaswen, Karen L. MacKenzie, W. Nicol Keith, Patricia Hentosh, Francis Rodier, Jiyue Zhu, Gary L. Firestone, Ander Matheu, Amancio Carnero, Alan Bilsland, Tabetha Sundin, Kanya Honoki, Hiromasa Fujii, Alexandros G. Georgakilas, Amedeo Amedei, Amr Amin, Bill Helferich, Chandra S. Boosani, Gunjan Guha, Maria Rosa Ciriolo, Sophie Chen, Sulma I. Mohammed, Asfar S. Azmi, Dipita Bhakta, Dorota Halicka, Elena Niccolai, Katia Aquilano, S. Salman Ashraf, Somaira Nowsheen, Xujuan Yang

**Affiliations:** a Life Sciences Division, Lawrence Berkeley National Lab, Berkeley, CA, United States; b Children's Cancer Institute Australia, Kensington, New South Wales, Australia; c University of Glasgow, Glasgow, United Kingdom; d Old Dominion University, Norfolk, VA, United States; e Université de Montréal, Montréal, QC, Canada; f Washington State University College of Pharmacy, Pullman, WA, United States; g University of California Berkeley, Berkeley, CA, United States; h Biodonostia Institute, Gipuzkoa, Spain; i Instituto de Biomedicina de Sevilla, HUVR, Consejo Superior de Investigaciones Cientificas, Universdad de Sevilla, Seville, Spain; j Sentara Healthcare, Norfolk, VA, United States; k Nara Medical University, Kashihara, Nara, Japan; l National Technical University of Athens, Athens, Greece; m University of Florence, Florence, Italy; n United Arab Emirates University, Al Ain, United Arab Emirates; o Cairo University, Cairo, Egypt; p University of Illinois at Urbana Champaign, Champaign, IL, United States; q Creighton University, Omaha, NE, United States; r SASTRA University, Thanjavur, Tamil Nadu, India; s The University of Rome Tor Vergata, Rome, Italy; t Ovarian and Prostate Cancer Research Trust, Guildford, Surrey, United Kingdom; u Purdue University, West Lafayette, IN, United States; v Karmanos Cancer Institute, Wayne State University, Detroit, MI, United States; w New York Medical College, Valhalla, NY, United States; x Mayo Clinic, Rochester, MN, United States

**Keywords:** Senescence, Telomerase, Oncogenic stress, p53, pRB

## Abstract

One of the hallmarks of malignant cell populations is the ability to undergo continuous proliferation. This property allows clonal lineages to acquire sequential aberrations that can fuel increasingly autonomous growth, invasiveness, and therapeutic resistance. Innate cellular mechanisms have evolved to regulate replicative potential as a hedge against malignant progression. When activated in the absence of normal terminal differentiation cues, these mechanisms can result in a state of persistent cytostasis. This state, termed “senescence,” can be triggered by intrinsic cellular processes such as telomere dysfunction and oncogene expression, and by exogenous factors such as DNA damaging agents or oxidative environments. Despite differences in upstream signaling, senescence often involves convergent interdependent activation of tumor suppressors p53 and p16/pRB, but can be induced, albeit with reduced sensitivity, when these suppressors are compromised. Doses of conventional genotoxic drugs required to achieve cancer cell senescence are often much lower than doses required to achieve outright cell death. Additional therapies, such as those targeting cyclin dependent kinases or components of the PI3K signaling pathway, may induce senescence specifically in cancer cells by circumventing defects in tumor suppressor pathways or exploiting cancer cells’ heightened requirements for telomerase. Such treatments sufficient to induce cancer cell senescence could provide increased patient survival with fewer and less severe side effects than conventional cytotoxic regimens. This positive aspect is countered by important caveats regarding senescence reversibility, genomic instability, and paracrine effects that may increase heterogeneity and adaptive resistance of surviving cancer cells. Nevertheless, agents that effectively disrupt replicative immortality will likely be valuable components of new combinatorial approaches to cancer therapy.

## 1 Introduction

Among the notable feats of evolution is the remarkable protection from cancer that is enjoyed by long-lived species such as humans. Despite billions of cell divisions and trillions of cells, humans remain, on average, cancer-free for more than 50 years. One of nature's notable tumor suppressive mechanisms is cellular senescence, a response to nonlethal stress that results in persistent cytostasis. In the absence of normal growth arrest accompanying differentiation, senescence imposes limits on the proliferative capacity of clonal cell lineages. Senescence can be induced by multiple stimuli, including intrinsic cellular processes such as telomere dysfunction and oncogene expression, but also by exogenous factors such as DNA damaging agents or oxidative environments. Abundant published evidence now supports the concept that senescence is a significant impediment to malignancy, and that it is ordinarily very stringent. Indeed, as a number of investigations have shown, many cell types in which one or more senescence pathway components are functionally inactivated remain susceptible to senescence – an indication that robust compensatory mechanisms exist for this important stress response. Despite the resiliency of the senescence response, however, it is prone to failure to varying degrees, depending upon genetic/epigenetic context. Failure of senescence in cells that have undergone oncogene activation, telomere dysfunction, and/or DNA damage can result in changes favoring malignancy and drug resistance. Elucidation of mechanisms that enforce senescence has been sought in expectation that such knowledge should lead to measures that prevent or reverse its failure in susceptible pre-malignant and malignant cell populations. In this review, we focus on telomeres and other mediators of senescence induction as candidate targets for the prevention and treatment of cancers.

## 2 Causes of senescence

Proliferating cells can respond to genotoxic and non-genotoxic stresses in a number of ways, including transient cell-cycle arrest, senescence, and cell death. Senescence is operationally broadly defined as a viable growth arrest characterized by the inability of affected cells to resume proliferation in the presence of appropriate mitogenic factors. While multiple cellular and molecular features, including increased cell size, accumulation of lysosomes, upregulation of cell cycle inhibitors, presence of senescence-associated heterochromatic foci (SAHF), and positive staining for senescence-associated beta-galactosidase (SA-βGal) activity, have been associated with senescent cells, no single feature is a universal and specific marker of senescence. Experimental and clinical evidence indicate that an intact senescence response is important for preventing unregulated growth and malignant transformation. In addition, the ability to undergo senescence can determine the efficacy of targeted cancer therapies. As described below, however, senescence is not a discrete mechanism or pathway that can be easily classified as either intact or entirely non-functional. Instead, it is a process that can result from many different inputs with degrees of sensitivity dictated by intrinsic as well as extrinsic factors.

### 2.1 Telomerase repression

In the absence of externally or oncogenically induced stresses, telomerase repression may be the only physiological impediment to indefinite replication. Replicative senescence, as originally described by Hayflick in cultures of cells from non-malignant tissues, is due to natural repression of telomerase and the resulting DNA damage response that occurs when the number of telomeric TTAGGG repeat sequences on the ends of chromosomes becomes too few to support the assembly of stable telomere complexes [1], [2]. Structures formed through interactions of TTAGGG repeat sequences with a protein complex referred to as shelterin function to “cap” the chromosome ends, protecting against DNA degradation, recombination, and chromosome fusion [3]. The telomeric TTAGGG repeats are replenished by telomerase [4], a ribonucleoprotein complex that consists of a catalytic reverse transcriptase protein subunit (hTERT, TERT) [5], [6], [7], an RNA template (hTR, TERC) [8], [9], [10], and other accessory proteins, including the RNA-modifying protein dyskerin [11], [12]. The presence of hTERT and hTR are the minimum requirements for recapitulation of telomerase activity *in vitro*. Telomerase activity and telomere length elongation in cancer are associated with up-regulation of both hTR and hTERT, while overexpression of hTR has been shown to boost telomerase activity and more dramatically extend telomere length in cells that express endogenous or ectopic hTERT [13], [14], [15]. Thus both telomerase components restrict telomerase activity and telomere length *in vitro*, illustrating the fact that both components are required for a functional telomerase holoenzyme. Although hTERT was initially considered as the limiting component of telomerase, evidence from biochemistry, promoter studies, mouse models, and human tumors has demonstrated contexts where hTR limits telomerase enzyme levels and telomere maintenance [13], [14], [15], [16], [17], [18]. At early embryonic stages, the hTERT gene and telomerase activity are expressed at high levels in many tissues [19], [20]. The hTERT gene then undergoes repression as embryonic cells differentiate into adult somatic cells [21]. From the neonatal period onward, hTERT transcripts and telomerase activity are nearly or completely undetectable in most human tissues [19], [22], [23], except in some highly proliferative tissues, such as lymphoid cells and tissue stem and progenitor cells [24], [25], [26], [27]. *In vitro*, attrition of TTAGGG repeats upon successive divisions in cells lacking sufficient telomerase activity ultimately results in DNA damage responses including growth arrest, followed by cell enlargement, chromatin condensation, and vacuolization – characteristic features of senescent cells. Multiple and distinct human cancer precursor lesions, but not corresponding malignant cancers, are composed of cells that display signs of telomere dysfunction-induced senescence [28]. Ectopic hTERT expression in many cell types prevents these senescent changes by stabilizing telomeres and extending replicative lifespan [29], [30], [31], [32]. While not intrinsically essential for malignancy [33], an extended lifespan or “immortalization” permits clonal cell lineages to accumulate rare genetic and epigenetic aberrations that together can cause malignant transformation.

In the absence of a telomere maintenance mechanism, telomeres shorten with each round of cellular replication to eventually reach critically short lengths that are unable to support stable formation of shelterin protein complexes that protect telomeres from DNA damage surveillance mechanisms [34]. In normal cells, dysfunctional telomeres and abnormal chromosomal structures initiate a p53-mediated DNA damage response [35]. The p16/pRB tumor suppressor pathway is also activated during replicative senescence in response to telomere dysfunction and DNA damage [36], [37]. Activation of these tumor suppressors halts cell cycle progression, initiates senescence, and prevents the propagation of abnormal chromosomes. However, genetic or epigenetic aberrations in p53 and/or p16/pRB pathways enable otherwise normal cells to continue to proliferate. Cells that bypass senescence as a result of defects in these pathways are subject to continued telomere shortening and telomere dysfunction with consequential evolution of complex karyotypes [38], [39], [40]. Critically short telomeres are fusogenic, resulting in formation of unstable structures, such as dicentric and ring chromosomes [38], [41]. Continued telomere dysfunction promotes the development of abnormal chromosomes through breakage-fusion-bridge (BFB) cycles, which are initiated by fused chromatids [40], [42]. During BFB cycles, fused chromatids form bridges during anaphase. Chromatin bridges break as cells continue through mitosis, resulting in uneven segregation of genetic material and unstable chromosome structures, which fuel further BFB events in daughter cells. BFB cycles continue until abnormal chromosome structures are lost or stabilized [43]. Resulting chromosome abnormalities include non-reciprocal translocations, deletions, gene amplifications, and whole chromosome losses [43]. These types of chromosomal aberrations have been demonstrated in association with telomere dysfunction in cell culture models, p53-null mice, tumor-derived cell lines, and pre-malignant conditions [38], [40], [42], [44], [45], [46], [47], [48], [49]. They are also the hallmarks of human carcinomas that feature chromosomal instability [46], [50], [51], [52], [53], [54]. By accelerating the rate and accumulation of molecular changes, telomere dysfunction-driven chromosomal instability may result in activation of telomerase, activation of oncogenes and/or silencing of tumor suppressor genes, which ultimately cooperate to promote malignant transformation, tumor progression, and drug resistance [44], [45], [46], [47], [48], [52], [54], [55].

### 2.2 Oncogenic stress

In contrast to replicative senescence, oncogene-induced senescence (OIS) often occurs independently of telomere status [56], [57], but shares many of the same morphological and biochemical features [36], [58], [59], [60], [61]. OIS is observed after ectopic expression of oncogenic RAS, and many of its effectors, including activated mutants of RAF, MEK, B-RAF, PI3K, AKT, and PIM [62], [63], [64], [65], [66], and other oncogenes such as CDC6, cyclin E and STAT5 [67]. Also, functional abrogation of tumor suppressors such as PTEN, pRB, Spn, and NF1, induces senescence-like responses [68]. Stress associated with aberrant DNA replication caused by these oncogenic changes appears involved in OIS [69], [70], [71], [72]. Although the mechanism is not fully understood, it may be due to increased expression of positive regulators of S-phase. Consequently, replicons refire or terminate prematurely, generating DNA breaks that initiate a DNA damage response and phosphorylation of p53 by DNA damage response kinases. Components of the MAPK cascade also increase the expression of the tumor suppressor ARF [73], and initiate a negative feedback loop that ultimately inhibits H/MDM2 [65], resulting in p53 stabilization.

The presence of senescent cells induced by oncogenic signaling has been documented in several precancerous tissues of both human and mouse, indicating that OIS occurs *in vivo* [74], [75], [76], [77], [78]. The first direct evidence of cellular senescence in a growth-arrested human neoplasm was reported in benign human melanocytic nevi (moles) [77]. Cells in nevi carry an activated oncogene product (B-Raf-E600), express elevated levels of the tumor suppressor p16, undergo long-term cell-cycle arrest, and display abundant SA-β-Gal activity. In addition, they do not show detectable signs of telomere erosion, suggesting that they have undergone OIS rather than replicative senescence. These and similar results reported by others [79] suggest that OIS in nevi acts as a barrier to melanoma development.

### 2.3 External agent-induced stress

Conventional genotoxic radiotherapy and chemotherapeutic regimens are potent inducers of senescence in cancer cells *in vitro* and *in vivo* (reviewed in [80]). Although this senescence response has been shown to involve many of the same DNA damage response mediators (*e.g.*, ATM, ATR, Chk1, Chk2) as those activated by telomere dysfunction and oncogene activation, it is noteworthy therapeutically that cancer cells lacking functional tumor suppressors such as p53 or pRB often retain the capacity to undergo external agent-induced senescence. While in some cases, senescence-inducing drugs clearly act through the generation of DNA damage, in other cases the mechanisms involve only alterations in DNA structure and function (*e.g.*, inhibition of DNA methylation or histone acetylation). Still other compounds may induce senescence through direct or indirect stimulation of stress sensitive kinases, creating imbalances in mitogen signaling pathways [81].

The chief clinical advantage of targeting senescence rather than outright cell death as a desired endpoint is that drug doses required to achieve cancer cell senescence are often much lower than doses required to achieve outright cell death. In prostate cancer cell lines, for example, a dose of 25 nM doxorubicin is sufficient to induce senescence, while doses >250 nM are necessary to cause cell death [82]. In practical terms, treatment with lower doses of genotoxic drugs that are sufficient to induce senescence but not cell death could provide equivalent or prolonged patient survival with fewer and less severe side effects. There are caveats to this approach, however, as mentioned elsewhere in this review. Chief among them are the questions of senescence reversibility and pro-tumorigenic paracrine effects of senescent cells that are not cleared by the immune system.

## 3 Mediators of senescence

Gene expression profiling has revealed that there is limited overlap among genes exhibiting altered expression in cells induced to undergo senescence by telomere shortening, oncogene induction, or external agents [83], [84], [85], [86], [87], [88]. The data indicate that fundamental differences exist in gene regulation during senescence activated by various signals in different cell types, despite similarity in the resulting phenotypes. Despite differences in upstream signaling, however, induction of senescence often involves convergent interdependent activation of tumor suppressors p53 and p16, the former protein initiating and the latter maintaining the response [89], [90]. While this sequence is demonstrable in lymphoma cells exposed to cyclophosphamide [91] and fibroblasts exposed to activated Ras [59], in other cases, such as human mammary epithelial cells exposed to suboptimal culture conditions [48], [92] and melanocytes which acquire B-RAF mutations [93], p16 activation and senescence occur independently of p53 activation. Although p53 and p16 are clearly involved in establishing senescence growth arrest, their precise roles in this process are incompletely understood.

### 3.1 p53

The importance of p53 in senescence was determined by inhibiting p53 function with dominant negative mutants, specific p53 antisense mRNA, oligonucleotides or viral oncoproteins (such as SV40 T antigen or HPV16 E6); such treatments were sufficient to substantially extend the lifespan of several cell types in culture [94]. Consistent with these findings, senescence is associated with the transactivation of p53 in cultured cells [95]. Coincident with telomere shortening and DNA-damage checkpoint activation, p53 is also activated *in vivo* [45]. Deletion of p53 attenuated the cellular and organismal effects of telomere dysfunction [45], [96]. Proteins that regulate p53 have also been implicated in senescence. MDM2 has p53 ubiquitin ligase activity and forms an autoregulatory loop with p53 [97]. Overexpression of MDM2 targets p53 for degradation and induces functional p53 depletion [98]. Expression of another factor upregulated in senescence, ARF, can release p53 from MDM2 inhibition and cause growth arrest in young fibroblasts [98]. Seeding mouse embryonic fibroblasts (MEFs) into culture induces ARF synthesis, which continues to accumulate until the cells enter senescence [99]. MEFs derived from ARF-disrupted mice [99] or wild type fibroblasts expressing an ARF antisense construct [100] are efficiently immortalized. Consistent with this observation, overexpression of MDM2 in MEFs produces efficient immortalization [100].

Senescence effects of p53 are mediated in part through increased expression of the CDK inhibitor p21^WAF1^ [101], [102], [103]. p21, in turn, prevents hyperphosphorylation and inactivation of pRB [104], [105]. In some human cells, elimination of p21 is sufficient to bypass senescence [101]. However, in MEFs, the absence of p21 does not prevent senescence [106], [107]. This finding indicates that at least one additional downstream effector can independently mediate p53-induced senescence. Candidate p53 effectors include 14-3-3 and GADD45, which inhibit G2/M transition, or p53-dependent transcriptional repression of c-Myc, which results in G1 cell cycle arrest [108]. Other signals may cooperate with p53 to induce senescence. For example, Ras-induced activation of PPP1CA, the catalytic subunit of PP1α, has been shown to be necessary for Ras-dependent senescence [109]. Independently of p53, PPP1CA can stabilize the active unphosphorylated form of pRB. In MEFs, E2F transcription factor associated repressor complexes are downstream targets of p53-induced proliferation arrest [110], indicating a convergence of the p53 and p16/pRB pathways at the level of E2F. However, in many human cells, inactivation of either p53 or pRB (*e.g.*, by viral oncoproteins or anti-sense oligonucleotides) independently and synergistically extends replicative life span [111], [112], [113]. These findings suggest that although the p53 and pRB pathways interact, they may also act separately to establish senescence.

### 3.2 p16/pRB

While the causes of p16 induction remain to be defined, its mechanism of action has been well characterized. The binding of p16 to cyclin dependent kinases (CDKs) 4 and 6 induces allosteric conformational changes that disrupt the interaction of these kinases with D-type cyclins [114], thus antagonizing activation of the CDKs. Through binding and inactivation of CDKs, p16 prevents phosphorylation and inactivation of pRB and similar “pocket” proteins, p107 and p130. This canonical description, while valid, obscures the differences in *RB* family functions that distinguish reversible cell cycle arrest from irreversible senescence-associated changes. Despite the similarities among *RB* family proteins, defects in pRB, but not in p107 or p130, have been associated with human cancers. This suggests that pRB has unique tumor suppressor properties not attributable to p107 or p130. In support of this concept, pRB has been shown to be preferentially associated with E2F targets involved in DNA replication during OIS, and suppression of pRB, but not p107 or p130, allowed continued DNA synthesis after induction of oncogenic RAS [115]. The pRB protein contains multiple phosphorylation sites and interacts with multiple protein complexes. It remains to be determined whether the spectrum of pRB dependent changes in a given cell type under specific conditions is simply determined by the duration of pRB activation or by qualitative differences in pRB modifications/binding interactions. Changes initiated by p16 expression are qualitatively and quantitatively distinct from those in cells undergoing transient pRB-dependent growth arrest. For example, in U2OS cells exposed to p16, pRB augments p130 at E2F-regulated promoters. Dean and co-workers [116] used chromatin immunoprecipitation (ChIP) assays to assess protein association with the E2F responsive cyclin E and A promoters. A 6-day induction of p16 resulted in a dramatic increase in pRB and E2F-4 associated with these promoters. Additional promoter-specific changes in the extent of binding to histone deacetylase HDAC1, SWI/SNF chromatin remodeling complex components BRG1 and Brm, and polycomb group protein HPC2 were noted.

**Fig. 1:**
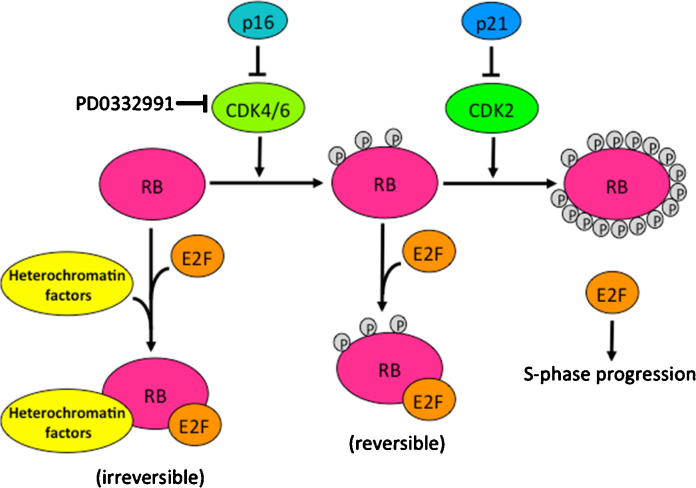
A simplified scheme is presented of hypothetical alternative phosphorylation states and growth arrest functions of RB family proteins. Gray circles represent phosphate groups added to RB family proteins by different cyclin-CDK complexes. The primary sites of action of endogenous CDK inhibitors, p16 and p21, as well as the small molecule inhibitor, PD0332991, are also shown.

Distinctions in pRB-associated phenotypes may be due to differences in the functionality of different phosphorylated forms of pRB (Fig. 1). Although growth factors are required for cyclin D1 synthesis, transiently growth-arrested cells often contain significant amounts of cyclin D3 associated with CDK4, and the level of CDK4 activity is sufficient for cell cycle progression if CDK inhibitors are removed [117]. Thus in transiently growth-arrested cells, pRB may be held preferentially in a hypophosphorylated rather than an unphosphorylated state. While many past studies have relied on the effect of hyperphosphorylation on the electrophoretic mobility of pRB to distinguish the hyperphosphorylated from the hypophosphorylated form, few have distinguished the unphosphorylated from the hypophosphorylated form [118]. E2Fs are more easily co-immunoprecipitated with the hypophosphorylated form of pRB than the unphosphorylated form of pRB in peripheral blood lymphocytes (PBLs) during early G1 [119]. Interestingly, transduction of p16 protein into PBLs leads to loss of pRB hypophosphorylation and loss of detectable pRB association with E2F-4. The lack of detectable association might be due to reduced affinity of the unphosphorylated form of pRB for E2F-4, or alternatively to relative insolubility of larger chromatin complexes containing both pRB and E2F-4. Regardless of the interpretation, the results suggest that pRB maintained in a minimally or completely unphosphorylated state in the presence of p16, is likely to have properties that differ from those of the hypophosphorylated form. Confirmation of this concept is apparent in the results of an expression profiling study of rat fibroblast cell lines [120]. In this study, the effects on global gene expression of a pRB allele refractory to cyclin-CDK phosphorylation (the presumed state of pRB in the presence of p16) were compared to those of E2F-2 overexpression. The targets of unphosphorylated pRB were consistent with genes downregulated by p16 overexpression, but showed lower correspondence to genes stimulated by E2F-2. Similar results using human U2OS cells were reported [121].

### 3.3 pRB-mediated heterochromatin formation during cellular senescence

pRB can play an active role in the formation of senescence-induced heterochromatic foci (SAHF) in human cells. Originally characterized in senescent fibroblasts [122], these foci consist of reorganized DNA and are enriched for proteins normally associated with heterochromatin, such as histone H3 methylated on lysine 9 (H3-K9Me), and heterochromatin protein 1 (HP1) proteins α, β, and γ. The formation of SAHF was slow, taking several days or weeks, depending on the initiating stimulus, and was reported to coincide with the enhanced association of E2F-target promoters with heterochromatin proteins. Notably, these changes were blocked by expression of the adenoviral E1A protein, which inactivates pRB and prevents senescence. Interestingly, senescent BJ fibroblasts, which poorly express p16 and were less stably arrested [122], displayed fewer SAHFs than senescent fibroblasts that express higher amounts of p16 [123].

The chromatin-based role of pRB in transcriptional repression is complicated, with multiple factors cooperating for transcriptional repression of specific promoters. For example, repression of cyclin A has been shown to be dependent on SWI/SNF chromatin remodeling, whereas other forms of transcriptional repression have been shown to be dependent on histone deacetylases or polycomb repressor components [116], [124], [125], [126]. In addition to the E2Fs, pRB can also associate with HP1 and histone methyltransferases such as SUV3-9H1, raising the possibility that pRB helps direct the process of histone methylation and HP1 recruitment to E2F responsive promoters during senescence. Consistent with this possibility, pRB showed colocalization with SAHFs in the nuclei of senescent cells, which was greater than that observed for p107 and p130 [122]. Additional work [127] has shown that p16-dependent repression by pRB at E2F target gene promoters involves the establishment of a stable repressor complex that is not displaced by the overexpression of E2F-1. Rather than displacing pRB, excess E2F-1 instead recruits more pRB, leading to direct transcriptional repression. In contrast, *Rb* family members, p130 and p107, which have not been demonstrated to be tumor suppressors, bind preferentially to target promoters in the absence of growth factors and in proliferating cells, respectively, and these repressor complexes are displaceable by E2F-1. HP1, which interacts with pRB, is associated with these distinct repressor complexes and follows a similar pattern of stability/displaceability. Efficient growth arrest by p16/pRB in MEFs is dependent on H3-K9Me, which provides a binding site for HP1. Differences in the stability of repressor complexes at promoters may underlie the different roles of pRB *versus* p130 and p107 in cell cycle regulation and tumor suppression [127].

Although the mechanisms responsible for formation and propagation of heterochromatin remain to be characterized, H3-K9Me, trimethylation of histone H4 lysine 20 (H4-K20Me), and recruitment of HP1 appear to be involved [128], [129]. Trimethylation of heterochromatic histone H3-K9 is accomplished by Suv3-9H1 and Suv3-9H2 [130], while Suv4-20H1 and Suv4-20H2 trimethylate histone H4-K20 [129]. pRB physically interacts with both these methyltransferase complexes, although the biological significance of these interactions remains unclear [131], [132]. Importantly, H3-K9Me was enriched at proliferation-associated gene promoters specifically in senescent cells, concomitant with the appearance of SAHFs, but not in quiescent cells [122]. In addition, Suv3-9, which is responsible for H3-K9Me, has been found to be required for OIS in murine lymphocyes, and for suppression of lymphoma [64]. Therefore senescent cells are thought to maintain growth arrest through the formation of heterochromatin at proliferation-promoting gene loci.

Molecular details of SAHF formation and stability remain to be determined. In addition to pRB, H3-K9Me, and HP1, SAHFs are known to be enriched in a histone H2A variant, macroH2A, previously associated with silenced chromatin [133], and HMGA proteins, which appear to be essential for SAHF formation [123]. Two evolutionarily conserved histone chaperones, HIRA and ASF1a, are also known to cooperate with pRB in the formation of SAHFs [134]. These proteins, human orthologs of proteins known to create transcriptionally silent heterochromatin in yeast, flies, and plants, may generate more extensive heterochromatin domains at positions designated by pRB. In WI38 human fibroblasts induced to undergo senescence by the introduction of an activated Ras oncogene, each chromosome condenses into a single SAHF focus [135]. The chromosome condensation is hypothesized to depend, in part, on increased nucleosome density due to HIRA/ASF1a-mediated nucleosome deposition. This chromatin condensation occurs prior to the accumulation of H3-K9Me and deposition of HP1 and macroH2A in chromatin, leading to speculation that HP1 proteins do not contribute to the acute onset of the senescent phenotype, but that instead, they might be required for the long-term maintenance of SAHF and the senescent state. Similarly, recent work using the same inducible OIS model has shown that the global pattern of repressive histone marks was largely unchanged during senescence, indicating that SAHF formation largely involves repositioning of chromatin bearing pre-existing marks rather than *de novo* formation of new marks [136]. This work also showed that H3-K9Me and H3-K27Me marks were not necessary for SAHF formation. However, the contribution of these individual components to senescence irreversibility remains to be determined.

## 4 Evidence that senescence is tumor suppressive

A protective role of senescence has been inferred in murine models of lung adenomas, T-cell lymphomas, prostate tumors, and pituitary tumors [74], [75], [76], [78]. In one example, Ras-V12 knock-in mice were shown to develop lung adenomas that were characterized by low proliferative indices, elevation of SA-β-Gal activity, and other senescence markers [137]. By contrast, the few adenocarcinomas that did emerge showed considerable proliferative activity and lacked senescence markers. In another example, Ras-V12-driven mouse T-cell lymphoma cells in which apoptosis was blocked entered senescence after drug therapy, and senescence was shown to be dependent on the chromatin-remodeling enzyme Suv3-9H1 [74]. In another study, p53 and p16 were found to cooperate in murine lymphoma cells, engaging a program of prolonged cell-cycle arrest in response to the alkylating agent, cyclophosphamide [91]. Mice bearing tumors capable of p16-induced senescence had a much better prognosis following chemotherapy than those harboring tumors with p16 defects. The study showed further that p16 loss and disruption of apoptosis by Bcl2 act independently to promote drug resistance. In a prostate tumor model, p53-dependent cellular senescence could be triggered by inactivation of the tumor suppressor, PTEN [75]. Similar findings have been reported in E2F-3 driven pituitary gland tumors [138].

Direct evidence that senescence is tumor suppressive has been generated using inducible murine models. In a model for p53-dependent liver cancer, the effect of p53 restoration was studied in established liver carcinomas [139]. Hepatoblasts expressing a conditional p53 shRNA produced invasive hepatocarcinomas. Soon after re-expression of p53, the tumors underwent dramatic regression associated with cellular senescence. The senescent cells acquired a specific gene expression profile that included upregulation of inflammatory cytokines; this led to activation of the innate immune system, which was responsible for clearing the tumor. This work established a link between the cellular senescence program and the innate immune system in suppressing tumorigenesis. Similar work by others using a mouse osteosarcoma model has also shown that re-expression of endogenous p53 leads to a senescence-like cell-cycle arrest and complete tumor regression [140].

Correlative data indicate that stress-induced senescence is tumor suppressive in human tissues as well. In malignant cells, senescence is a well-documented consequence of various chemotherapeutic regimens [80]. In a specific example in which SA-βgal activity was used as a marker of senescence, 15/36 breast tumor resections after neoadjuvant chemotherapy (cyclophosphamide, doxorubicin, and 5-fluorouracil) were found positive for SA-βgal, while only 2/20 untreated tumors showed any SA-βgal staining, suggesting that the induction of senescence may be favorably related to treatment outcome [141]. The senescence response was associated with those tumors bearing wild-type p53 alleles and exhibiting p16 expression. Interestingly, the normal tissue of chemotherapy-treated patients was completely negative for SA-βgal, indicating potential selectivity in the senescence response. Similar observations have been made in lung tumors resected from patients receiving neoadjuvant chemotherapy [142].

## 5 Evidence that senescence can promote malignancy – the senescence associated secretory phenotype

Unlike cells that are removed by other tumor suppressor mechanisms such as programmed cell death, senescent cells can persist and continue to actively interact with surrounding cells and tissues for extended periods of time. Defective elimination of senescent cells may lead to unregulated accumulation in aging tissues and at sites of age-associated pathologies including cancer [75], [86], [93], [143], [144], [145], [146], [147], [148], [149], [150], [151], [152], [153], [154]. Experimental removal of accumulated p16 positive senescent cells in a progeroid mouse model was sufficient to reverse some age-associated pathologies, demonstrating that accumulation of senescent cells is responsible for some tissue dysfunctions [155]. In the context of tissue repair, current evidence suggests that senescent cells are normally eliminated in a process that involves immune system functions [156], [157], [158], [159], [160], [161]. Whether to stimulate tissue repair or their own elimination, senescent cells actively interact with their tissue microenvironment, possibly through direct cell–cell contacts, and undoubtedly using paracrine signals (secretion). Protein secretion by senescent cells has been collectively termed the Senescence-Associated Secretory Phenotype (SASP) [162]. Current data support the idea that senescent cells interact with and modify their microenvironment using this secretory program. Signaling elements regulating the SASP include the DNA damage response (DDR), p38MAPK, the JAK/STAT pathway and transcription factors such as NF-κB and C/EBPβ [163], [164], [165], [166], [167], [168].

**Fig. 2:**
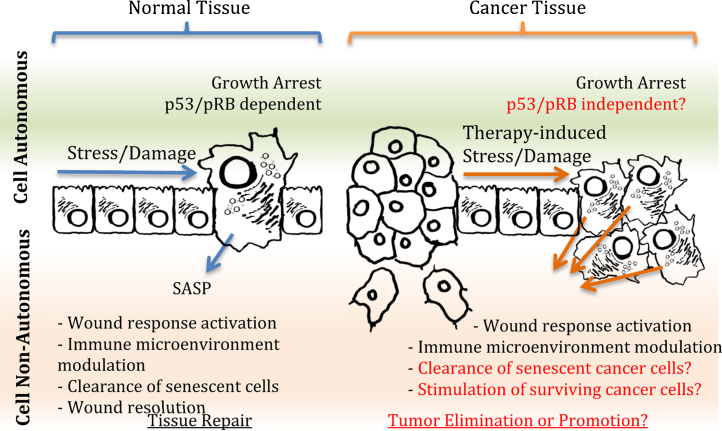
The senescence associated secretory phenotype (SASP) aids in the clearance of senescent cells, but can potentially promote proliferation of tumor cells that are not stably growth arrested. Cell-autonomous growth arrest associated with senescence prevents the proliferation of damaged cells and is at least partially dependent on p53/pRB pathways. In normal tissues, senescence also results in the activation of non-autonomous secretory factors that participate in wound response signaling, culminating in senescent cell clearance and tissue repair (left panel). In cancer tissues (right panel), activation of non-autonomous secretory factors can be altered and/or increased by treatment with therapeutic agents. However, cancer cells often harbor compromised p53/pRB pathways, and as a result, growth arrest may not occur or may be less stable. Alterations in the types or abundance of secretory factors released by such cells may interfere with immune clearance and/or stimulate the growth of nearby cancer cells that have escaped cell death. Important questions regarding the impact of senescence in the context of cancer therapy are highlighted in red.

The SASP was originally documented indirectly through the ability of senescent cells to influence the biology of other cells [169]. Subsequently, mRNA profiling, targeted proteomics (antibody arrays), and genome-wide RNA interference screens have been used to characterize SASP factors and their effects [163], [164], [170], [171], [172], [173], [174]. Multiple SASP factors have been directly validated for their ability to modulate the biology of senescent cells or that of surrounding cells [175], [176], [177]. While individual SASP components may vary depending on cell type and context, a few ubiquitous SASP factors have emerged, including pro-inflammatory IL6 and IL8, extracellular matrix remodeling MMP3, and growth promoting GroA [170]. The SASP supports positive aspects of cellular senescence such as growth arrest and proper tissue repair (Fig. 2). Autocrine activities include reinforcement of p53-dependent growth arrest *via* cytokine signaling loops [163], [164]. Paracrine activities include orchestrating the activity of specialized repair cells, including activated stellate cells, fibroblasts and immune cells, which are responsible for the resolution of wound responses [156], [157], [159]. The SASP is also believed to modulate the clearance of senescent cells by the immune system [157], [159].

In some contexts, however, presumably when senescent cells cannot be properly cleared by the immune system, negative consequences can occur [153], [178], [179], [180]. Senescent cells may gradually accumulate and displace normal cells, rendering affected tissues dysfunctional. Senescent cells may also modify the local microenvironment making it more supportive of survival and/or growth of potential pre-neoplastic cells. Following cancer therapy, surviving cells exhibiting the SASP could create a protective microenvironment, or a niche, for subsets of cancer cells that can initiate cancer recurrence. Cancer promoting effects of specific SASP factors have been shown *in vitro* and *in vivo*. For example, MMP3 secretion by senescent cells perturbed the proliferation and differentiation of cultured normal breast epithelial cells [181] while secretion of HGF, AREG, or GroA increased proliferation of cultured cancer cells [173], [174], [182]. Cytokines IL6 and IL8 promoted invasiveness and epithelial to mesenchymal transition (EMT) of established cancer cell lines [173]. In xenograft models, senescent cells accelerated the growth of weakly tumorigenic human cell lines [169]. This was attributed to MMP3 [183], but may also involve other MMPs secreted by senescent cells [160]. Secretion of VEGF may be responsible for increased vascularization observed in xenograft models incorporating senescent cells [184]. Additional data support a role of the SASP in cancer recurrence. For example, IL6 secretion by senescent cells in post-chemotherapy murine thymus was sufficient to create a protective niche for subsets of lymphoma cells [167], [185]. Similarly, SASP factors were linked to recurrence of chemotherapy treated murine mammary cancers [186]. Finally, in another murine cancer model, secretion of SASP factor WNT16B by senescent stromal cells supported continued growth of prostatic epithelial cancer cells after chemotherapy [187]. Importantly, increased WNT16B levels were also detected in post-therapy human prostate, ovarian, and breast cancers.

## 6 Determinants of senescence stability

Contrary to popular belief, the senescence growth arrest is not always irreversible. Since senescence is a response to stress, genomic and epigenomic aberrations that ameliorate stress (*e.g.*, telomerase activation) or compromise cellular ability to sense or transduce stress-related signals (*e.g.*, p53 inactivation) have the potential to promote immortalization and resistance to therapeutically induced senescence. Evidence obtained using cell sorting and videomicroscopy showed that fully senescent non-malignant human keratinocytes are capable of spontaneously yielding mitotically competent progeny [188]. Similarly, fluorogenic tracer and video microscopy were used to show that morphologically senescent cells in cultures of human mammary epithelial cells expressing the NeuT oncogene are capable of dividing [189]. In addition, fully senescent human fibroblasts and human mammary epithelial cells could be stimulated to resume proliferation by inactivating p53 – provided the cells had not expressed p16 [122]. Human mammary epithelial cells lacking the ability to express functional p16 are prone to unstable growth arrest and chromosomal rearrangements during telomere dysfunction-induced senescence [48]. The genomic instability generated by telomere dysfunction can complement pre-existing genetic aberrations to yield immortal cells [190], [191]. Cells that emerge from such cultures display gross genetic aberrations, many of which are also seen in human cancers [54], suggesting that the same molecular lesions that enable escape or circumvention of senescence *in vitro* occur during oncogenesis *in vivo*.

Determinants of the stability of the senescence response have not been well characterized. Yet, the implications of delayed cell cycle re-entry or permanent senescence for patient prognoses are profound. In the former case, transient senescence may allow tumor cells time to repair or accommodate the chemotherapeutic stress (*e.g.*, damaged DNA), effectively conferring drug resistance [192]. Conversely, therapeutic efficacy would be enhanced by enforcement of stable senescence in cancer cells that manage to retain viability in the face of a therapeutic challenge. The mechanisms determining these exclusive outcomes are poorly understood, although clinical and experimental data indicate that the status of p21 and p16, and associated tumor suppressors (*e.g.*, p53, pRB) play critical roles.

The reversibility of p16-induced growth arrest has been examined in human U2OS cells in which the transcription of an exogenously introduced p16 gene was regulated by tetracycline [193]. Induction of p16 for one day arrested most cells in the G1 phase of the cell cycle; if the inducer was then removed, p16 levels returned to baseline and growth resumed within 3–5 days. If, however, p16 was induced for 6 days, DNA synthesis remained strongly inhibited and the cells acquired morphological features of senescence. These results demonstrated that sustained p16 expression is sufficient to impose a stable block to cell proliferation that becomes independent of p16 expression after a defined period of time.

In another model employing breast cancer cell lines stably expressing shRNAs against each of the individual RB family proteins, p16 induction still resulted in irreversible G1 growth arrest in each case [194]. This finding suggests that there is some redundancy in the ability of the individual RB family proteins to mediate irreversible growth arrest. Cases of functional redundancy within this gene family have been reported in a number of murine and human cell types [195], [196]. This finding may explain why aberrations in upstream regulators such as p16, cyclin D1 and CDK4, which presumably affect the regulation of all three RB family proteins simultaneously, are more common than aberrations in the individual RB family proteins themselves in some cancers. From a clinical standpoint, it is encouraging that even aggressive cancer cells lacking both p53 and pRB tumor suppressors, are susceptible to induction of irreversible senescence. This suggests that therapies employing small-molecule inhibitors of CDK4/6 may be effective even in some tumors lacking functional pRB.

A representative small molecule CDK inhibitor that has recently been approved for clinical use is PD0332991 (Palbociclib; Supplementary Table 1). This drug has been tested against 39 individual serine, threonine, and tyrosine kinases, representing most of the primary protein kinase families, and has shown highly selective inhibition of CDK4 and CDK6 [197], [198], [199]. Importantly, oral PD0332991 administration alone at doses that were well tolerated by host animals was sufficient to cause regression of a variety of human tumor xenografts [197]. The mechanism by which this small molecule causes tumor regression in xenograft studies has been unclear, since in short-term cell culture studies (72 h) it has been shown to cause cytostasis rather than cytotoxicity [197]. Recent assessment of PD0332991 using short-term growth assays indicated that it was effective in inducing growth arrest of many estrogen receptor (ER) positive breast cancer cell lines, but that a number of ER(−) lines were able to maintain pRB phosphorylation and proliferation in its presence [200]. Several of the ER(−) cell lines exhibiting resistance to the CDK4/6 inhibitor retained phosphorylated pRB in the presence of the CDK4/6 inhibitor. This indicates that another kinase, most likely CDK1 or CDK2, was capable of phosphorylating and inactivating pRB in the absence of CDK4/6 activity in these cell lines. Thus, in many ER(−) breast cancer cells, targeting of CDK1 or CDK2 instead of, or in addition to, CDK4 may be required to initiate senescence. However, if the resulting senescence response is not stable, this may be clinically counterproductive. Indeed this danger was illustrated in a recent report that showed CDK4/6 inhibition protected ER(−) breast cancer cells from doxorubicin-mediated cytotoxicity [201].

Supplementary table related to this article can be found, in the online version, at doi:10.1016/j.semcancer.2015.03.007.



## 7 Defects in telomerase regulation

Telomerase is almost universally re-expressed in cancer cells and is regulated at multiple levels involving genetic, epigenetic, transcriptional, post-transcriptional, post-translational, and sub-cellular shuttling mechanisms. At the genetic level, both hTR and hTERT genes have been reported to be amplified in some tumors. Increased hTR gene copy number was detected frequently in head and neck and cervical carcinomas [202] while hTERT gene amplifications have been found in primary cancers and cell lines, including those of brain, breast, cervix, liver, and lung [203], [204], [205], as well as primary and metastatic melanomas [206]. In most cases, the amplified region encompassed most or all of chromosome 5p [13], [203], [206]. However in several cases, chromosomal break points were mapped to regions close to the hTERT promoter, suggesting that chromosomal rearrangements could either relieve the promoter from its stringent repressive epigenetic environment or place it in the proximity of enhancers at different chromosomal sites [207]. Genetic polymorphisms may also contribute to variations in telomere length and cancer development. In a recent genome wide association study, single nucleotide polymorphisms (SNPs) in seven loci were found to be associated with differences in mean leukocyte telomere length and risks of cancer and other age-related diseases [208]. These loci corresponded to genes encoding hTERT and hTR, as well as other proteins involved in telomerase complex assembly and telomere maintenance. SNPs in multiple regions of the hTERT locus, including the promoter and downstream introns, were shown to be associated with telomere length and risks of various malignancies, including breast, ovarian, and prostate, glioma, lung, and urinary bladder cancer [209], [210], [211].

Both the hTR and hTERT genes are subject to tumor specific epigenetic regulation. H3-K9Me marks were increased at the hTR promoter in normal fibroblasts and in several ALT cell lines with low hTR expression in comparison with telomerase positive cancer cell lines, suggesting a role in hTR suppression, while H3-K4Ac, H4-K4Ac, H3-K9Ac and H3-K4Me were associated with hTR expression. Similar analysis of the hTERT promoter also revealed H3-K9Me associated with transcriptional suppression and higher levels of H3-K4Ac, H4-K4Ac and H3-K9Ac in cancer cells compared with normal fibroblasts, though H3-K4Me was not strongly associated with expression in the panel tested [212].

In cancer cells lacking chromatin mediated silencing present in normal cells, both genes are subject to regulation by a wide variety of distinct, but overlapping, transcription factors. hTR is generally expressed at low levels in normal cells and is substantially up-regulated in cancer cells as shown both by fluorescence *in situ* studies of a wide range of tumor samples [213] and by tumor specific expression of hTR promoter-driven transgenes in the setting of gene therapy studies [214], [215]. The hTR gene promoter is activated by NF-Y, Sp1, pRB and HIF1, and is suppressed by Sp3, MDM2 and active JNK signaling which causes a switch from Sp1 to Sp3 binding at the endogenous promoter [216], [217], [218]. However, beyond these findings, relatively little attention has been paid in the literature to hTR transcriptional mechanisms.

**Fig. 3:**
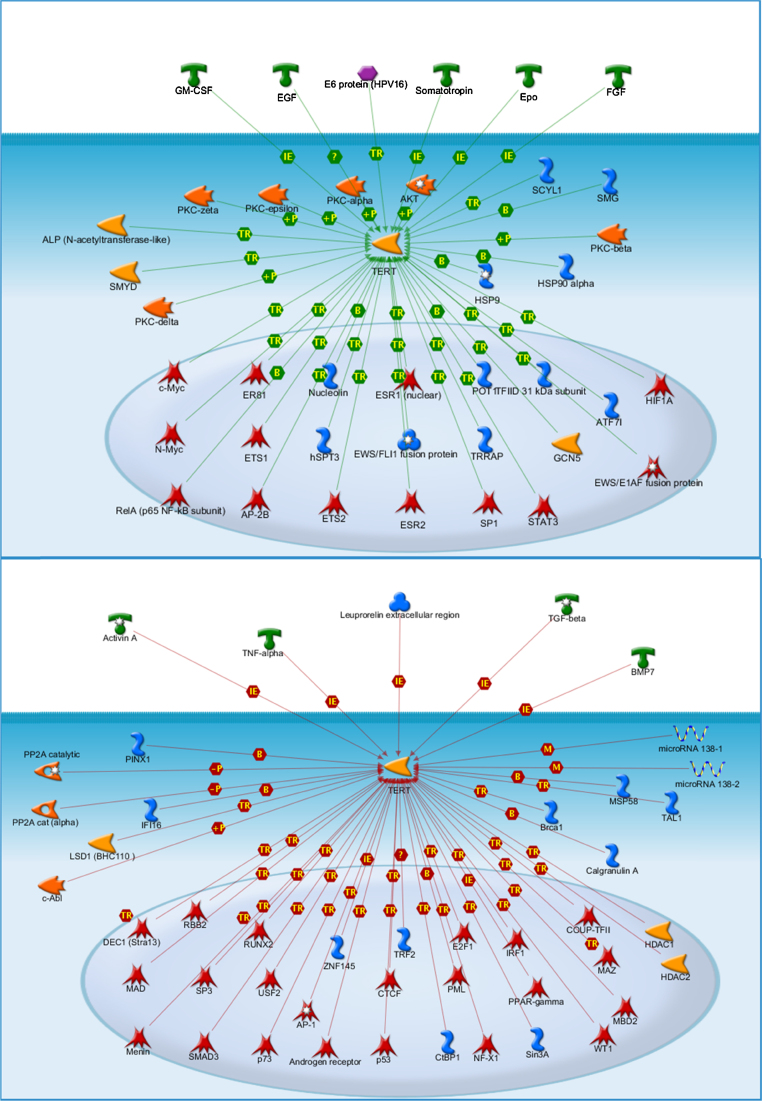
Mediators of hTERT activation and repression. Green arrows (top panel) indicate reported interactions that activate, while red arrows (bottom panel) indicate reported interactions that inhibit hTERT. Letters indicate mechanism (TR, transcriptional regulation; B, binding; -/+P, de/phosphorylation; IE, influence on expression).

In contrast, the hTERT gene has been intensely studied since its molecular cloning in 1997 (Fig. 3). hTERT transcripts are nearly or completely undetectable in most normal cells, but are expressed at low levels which are sufficient to drive telomere maintenance in cancer cells. Substantial work has focused on the core promoter region, which is sufficient for tumor specific activity. This region contains a number of binding sites for known transcription factors including c-Myc, HIF1, ETS, E2F and Sp1/Sp3, which integrate hTERT transcriptional responses with a number of important pathways that are dysregulated in various tumor types [219], [220], [221]. Interestingly, HIF1 is also involved in post-transcriptional regulation of hTERT splicing [222].

In general, oncogenic growth promoting pathways have usually been found to activate telomerase expression and promoter activity, while pathways controlling growth suppression, cell death and senescence have the opposite effect. For example, growth factor signaling through MAPK pathways increases hTERT expression in part *via* ETS factors [223]. Interestingly, recent studies have shown that telomerase can be activated by point mutations in the hTERT core promoter occurring with particularly high frequency in both familial and sporadic melanoma samples [224], [225]. These mutations occurred more frequently than BRAF or NRAS mutations and each generated a binding site for ETS family transcription factors, including the TCF subgroup activated by MAPK and BRAF signaling.

Other hTERT activation pathways that are frequently deregulated in various cancer settings include those influenced by CDK2 and CDK4 [226] and AKT [227], whereas deregulated repression pathways include those influenced by TGFβ [228], TNFα [229], and other cell cycle inhibitors [230]. Disrupted developmental pathways can cause hTERT expression. For example, the hTERT gene has been found to be a direct target of the Wnt signaling [231], [232], [233]. The Wnt signaling pathway is known to play essential roles in development and stem cell renewal. Binding of Wnt ligand to its receptor Frizzled activates GSK3β kinase, which blocks ubiquitin-dependent degradation of β-catenin. Stabilized β-catenin enters the nucleus, forms a complex with LEF/TCF, and activates a set of target genes including c-Myc. Myc protein binds to an E-box element in the hTERT core promoter and activates hTERT transcription. In addition, β-catenin can also form a complex with transcription factor Klf4 and bind to the hTERT promoter directly [231].

Recently, a whole kinome siRNA screen in ovarian cancer cells for regulators of the hTERT promoter revealed at least 68 kinases that participate in pathways upstream of hTERT regulatory transcription factors, underscoring the complexity of the signaling environment [234]. Hence, it is perhaps more useful and realistic to consider hTERT regulation in a systems context as a dynamic network, in which cell-specific mechanisms are likely to come into play, than to focus on individual factors. For example, while c-Myc has been shown to activate hTERT transcription in cancer cells and some normal cells [235], moderate overexpression of c-Myc by itself is not sufficient for activation of endogenous hTERT genes in normal human mammary epithelial cells [191]; in the latter case, additional genomic alterations were needed for telomerase activation and cellular immortalization. Many other factors bind the extended hTERT promoter region, co-operating with those at the core promoter and with upstream pathways. Different combinations of transcription factors may activate telomerase expression in specific cancer cells. Given this complexity, there is clearly a need for approaches to study telomerase regulation at the systems level. A possible positive implication of the complexity is that, if sufficiently well understood, it could lead to precise therapeutic ablation of telomerase expression in cancer cells through combinatorial targeting of cooperating factors in specific cancer cell contexts.

## 8 Therapeutic targeting of telomerase

Intrinsic differences in telomere maintenance between normal and cancer cells provide an attractive therapeutic opportunity. Several direct strategies to exploit the dependence of cancer cells on aberrant telomerase expression for telomere homeostasis and immortality have been reported. The key advantages of targeting telomerase in comparison with most other cancer targets are its relative universality, criticality and specificity for cancer cells, including putative cancer stem cells [236]. Approximately 90% of human cancers and virtually all adenocarcinomas display significantly higher levels of telomerase compared to normal cells, thereby implicating telomerase as an intriguing target of potential anticancer therapeutics [22]. No other tumor-associated marker is as widely expressed. Moreover, telomerase, encoded by non-redundant genes, is the most efficient known mechanism for maintenance of telomeres and replicative immortality. Although a telomerase-independent alternative lengthening of telomeres (ALT) mechanism exists for telomere maintenance in cell lines and cancers in which telomerase is not active or is suppressed [237], [238], some studies suggest that ALT cells are not as biologically robust as telomerase-positive cancer cells, and may have heightened susceptibility to drug regimens that induce oxidative stress [239], [240]. Thus cancers may be less likely to develop resistance to telomerase-based therapies than to other targeted therapies whose targets may be compensated for by functionally similar proteins and pathways. In addition, the low or transient expression of telomerase in normal tissues, including normal stem cells, and the generally longer telomeres in normal stem cells *versus* cancer cells, provide degrees of specificity to telomerase-based drugs and reduce the probability of toxicity to normal tissues [241]. All of these factors suggest that cancer drugs based on telomerase might have a high therapeutic index.

Many distinct classes of anticancer compounds directed toward telomerase can conceivably be developed because of the varied cellular processes that regulate telomerase expression and activity. For example, cellular telomerase activity can be controlled through changes in gene transcription and alternative splicing of the telomerase components, and by the nuclear translocation, phosphorylation, folding and turnover of individual components that lead to and regulate the rate of telomerase complex assembly and accessibility to telomeres [242], [243]. The therapeutic potential of targeting telomerase-mediated telomere maintenance in cancer cells was first demonstrated by expression of a dominant negative mutant form of hTERT (DN-hTERT) in tumor-derived cell lines [31], [244]. These studies showed that inhibition of telomerase in solid tumor and leukemia cell lines induced progressive telomere shortening and eventual proliferative arrest or cell death *via* apoptosis. They also demonstrated that expression of DN-hTERT inhibited anchorage independent growth and impeded the development of malignancies in xenografted mice [245], [246], [247], [248]. The inhibitory effects of DN-hTERT were also experimentally demonstrated using primary AML cells *in vitro* and *in vivo* using a murine model [248].

### 8.1 Oligonucleotide inhibitors

Studies that utilized antisense oligonucleotides, including chemically modified nucleic acids (PNA) that target hTR, provided proof-of-principle evidence of the effectiveness of this approach as a means of specifically inhibiting telomerase and inducing telomere shortening [249], [250], [251], [252], [253], [254]. In human tumor cell lines, 2′-O-MeRNA, phosphoramidate and PNA oligomers induced telomere shortening with the subsequent onset of apoptosis over long-term culture periods [252], [254], [255], [256]. In particular, a N3′–P5′ thio-phosphoroamidate oligonucleotide targeted to the template region of human hTR (GRN163) was shown to have efficacy against multiple myeloma and non-Hodgkin's lymphoma cell lines, as well as activity toward patient-derived cells [256], [257], [258]. The specificity of this compound was evidenced by telomere shortening, and by the relatively high sensitivity of multiple myeloma cells with short telomeres in comparison to cells with longer telomeres [257], [258]. A lipid conjugated form of GRN163, referred to as GRN163L or imetelstat, exhibited improved cell uptake and was shown to more effectively inhibit telomerase, cause more rapid telomere shortening and thus elicit more rapid growth arrest than the non-lipidated compound [259], [260]. Although one study reported that imetelstat caused cultured mesenchymal stem cells (MSCs) to arrest in G1 phase of the cell cycle, the MSCs were able to resume growth after imetelstat was removed [261]. Therefore, short-term imetelstat exposure did not appear to induce senescence or cell death in MSCs. The efficacy of imetelstat against cancer cells was demonstrated in a variety of preclinical models, including mice xenografted with human cell lines derived from liver, breast, lung and prostate cancers, as well as multiple myeloma [259], [262], [263], [264], [265]. Together, the preclinical studies of imetelstat validated targeting the template region of hTR as an effective approach to telomerase inhibition. However, evidence also emerged from these studies suggesting that in addition to inhibiting telomerase, imetelstat also has off target effects that disrupt the cytoskeleton and alter adhesive properties of tumor cells [266], [267]. Inhibition of breast and lung cancer metastases in an animal model was attributed to this telomerase-independent action of imetelstat [266].

Following the success of the preclinical investigations of imetelstat, phase 1 clinical trials were initiated in which imetelstat was tested as a single agent in patients with various aggressive liquid and solid tumors (Supplementary Table 1). The limited information that was released following the completion of those studies suggested that imetelstat presented minimal adverse effects, with thrombocytopenia and neutropenia being the dose limiting toxicity [236] (http://www.geron.com/imetelstat). Subsequent phase II trials have focused on hematologic disease, childhood CNS malignancies and the application of imetelstat in maintenance therapy for non-small cell lung cancer and metastatic breast cancer patients previously treated by standard chemotherapy and surgical debulking. Data emerging from these trials suggest limited benefit in these specific clinical settings. Disappointing progression-free survival results from a phase 2 trial of imetelstat as maintenance therapy following platinum chemotherapy in patients with non-small cell lung cancer have led the Geron Corp. to suspend further development of imetelstat in solid tumors. However, it has been argued that results from the lung cancer trial showed improved outcomes for a small subset of patients with tumors that had short telomere lengths. Among the trials for hematologic disorders, encouraging data from 13 patients treated with imetelstat for essential thrombocythemia were presented at the annual meeting of the *American Society for Hematology* in December 2012. The ability of imetelstat to reduce platelet counts in essential thrombocythemia is consistent with earlier trials that reported thrombocytopenia as an adverse effect.

While imetelstat, targeting hTR, was the first telomerase inhibitor to undergo trials in patients, modified antisense oligonucleotides targeting hTERT mRNA have also been shown to impede the proliferation of tumor cells. However, in contrast to the lag period observed in preclinical investigations of imetelstat, the proliferative defect induced by hTERT inhibition was immediate, and occurred in the absence of apparent telomere shortening [268], [269], [270]. A modified oligonucleotide directed toward hTERT mRNA was also shown to sensitize leukemic cell lines and primary cultures established from AML and CML patients to the drug cis-diamminedichloroplatinum. Ribozymes and small interfering RNAs (siRNAs) targeting hTERT have also been employed to suppress telomerase activity, impede proliferation, and sensitize tumor cells to cytotoxic drugs [271], [272], [273], [274], [275]. The potent anti-proliferative effects that have been demonstrated in tumor cells depleted of hTERT are consistent with a body of evidence that describe telomere-length independent functions of hTERT and gene expression changes induced by repression of hTERT [276], [277], [278], [279], [280], [281]. These observations further highlight the potential benefit of direct therapeutic targeting of hTERT, although the translation of these findings to the clinic await the development of new technologies for efficient delivery of siRNA and ribozymes to tumor cells.

### 8.2 Small molecule telomerase inhibitors

Small molecular weight compounds that inhibit telomerase activity have been identified in screens of chemical libraries or synthesized based on the structure of the tea catechin, epigallocatechin-3-gallate (EGCG; see Section 8.4), which is a known naturally occurring telomerase inhibitor [282], [283], [284], [285], [286], [287]. Small molecular weight telomerase inhibitors characterized to date belong to a range of chemical classes and include 2-[3-(trifluoromethyl)phenyl]isothiazolin-3-one (TMPI), Rhodacyanine (FJ5002), N-[3-[(2,3-dihydroxybenzoyl)amino]phenyl]-2,3-dihydroxybenzamide (MST-312) and (E)-2-(3-(naphthalene-2-yl)but-2enamido)benzoic acid (BIBR1532). BIBR1532 is one of the more extensively used inhibitors and was shown to induce telomere shortening, impede proliferation of tumor cell lines *in vitro* and limit tumor formation in xenografted mice [284], [288], [289]. At a low concentration (10 μm), BIBR1532 had no effect on short-term proliferation or survival, whereas higher concentrations (50–80 μm) were acutely cytotoxic. Notably, toxicity was also observed when telomerase-negative leukemia cells were treated with a high concentration of the BIBR1532. The cytotoxic effects of BIBR1532 were attributed to telomere uncapping, however non-telomeric effects have not been ruled out. Limited bioavailability of this compound has prevented its translation to clinical trials in patients.

Other indirect approaches to targeting telomerase can and are being considered. A range of pathways and mechanisms may be tractable for such inhibition. For example, the PI3K-Akt-mTOR pathway regulates cell size, progression of the cell cycle, and cell survival, and is considered a master regulator of protein synthesis [290]. mTOR – a serine/threonine kinase – is frequently dysregulated in cancer cells [291]. An association between mTOR and telomerase activity has been shown using the prototypical mTOR inhibitor rapamycin that, in addition to its other effects, causes inhibition of telomerase activity [292], [293], [294], [295], [296]. Some phytochemicals (see below) act in a similar manner to rapamycin with respect to telomerase inhibition [294], [297].

### 8.3 Immunotherapeutic approaches

Studies that demonstrated telomerase antigens on the surface of tumor cells provided impetus for pursuing telomerase immunotherapy as a therapeutic approach to the treatment of the broad spectrum of human malignancies that express telomerase [298], [299], [300]. Following preclinical studies of telomerase immunotherapy for treatment of leukemia and other malignancies [301], [302], immunotherapy products were developed for use in telomerase vaccination clinical trials [236], [303] (Suppl. Table 1). Indeed one product, GV1001, has progressed to phase III clinical trials, in which it is being used in combination with gemcitabine for the treatment of pancreatic cancer [236]. Concerns that telomerase immunotherapy may be detrimental to normal hematopoietic progenitor cells were somewhat allayed by investigations that showed there was no significant reduction in the frequency of clonogenic progenitor cells or NOD/SCID repopulating cells within the bone marrow of cancer patients after vaccination [304]. In accordance with this result, limited hematologic toxicity, such as grade I anemia and thrombocytopenia, were reported to occur during telomerase vaccine trials [236].

### 8.4 Telomerase-directed gene therapy

The general aim of most tumor specific gene therapy is to selectively kill cancer cells while leaving normal cells unharmed by expressing high concentrations of a therapeutic protein only in malignant cells. Transcriptional targeting, in which a therapeutic gene is placed under transcriptional control of a tumor-specific promoter, is a potentially powerful tool to achieve this aim. Although their activities are quite different, the promoters for hTERT and hTR are attractive candidates for use in gene therapy since they are both active in the vast majority of cancer cells tested [17]. Most telomerase gene therapy strategies that have been tested can be broadly categorized as cytotoxic gene therapy or oncolytic virotherapy approaches, both of which aim directly to kill cells expressing telomerase while sparing normal cells that do not, thereby circumventing the issue of the phenotypic lag in cells in which telomerase activity is merely blocked [17]. Using these gene therapy approaches, cell lines covering most of the major common malignancies have been targeted effectively *in vitro*. Critically, almost all of these studies have shown specificity using normal cell strains. Efficacy against xenograft models has also been repeatedly shown across multiple tumor cell types. Although there is a concern that the activities of the core telomerase promoters in normal mouse tissues may not reflect their activities in human, several groups have shown that systemic delivery of telomerase-specific gene therapy constructs does not result in significant off-target liver toxicity. In addition, the biodistribution of systemically delivered hTR and hTERT-specific transgene expression has been investigated by imaging and reporter assays; activity appears to be low or absent in normal tissues (for a review of efficacy and selectivity data, the reader is referred to tables in [17]). Therefore, proof-of-principle broad spectrum, selective targeting by the telomerase promoters has been convincingly shown in preclinical models.

### 8.5 Phytochemicals

Attractive anticancer agents due to their low cost and accessibility in diets, phytochemicals may have selective telomerase-inhibiting and senescence-inducing properties. Experimental evidence obtained in cultured human cancer cells suggests that a variety of natural phytochemicals from dietary and non-dietary sources represent promising sources of chemotherapeutic agents that can potentially target telomerase with few side effects (reviewed in [242], [297], [305]). Regulation of telomerase activity and/or expression, localization of specific components of the telomerase protein–RNA complex or posttranslational modifications by phytochemicals have been observed. Among the first phytochemicals reported to repress telomerase was the compound berberine, leading to identification of the more potent anti-telomerase analogue FJ5002 [282], though this was not further developed. Indicative of the complexities of understanding the precise mechanism of action of natural products, tested phytochemicals appear to disrupt different telomerase-associated processes in cancer cells from different tissue sources [242]. Also, a few studies have shown that certain phytochemicals can inhibit or stimulate expression of telomerase components in different cancer cells [242]. Because of the varied effects on telomerase, specific sets of phytochemicals could conceivably be used alone or as complementary agents in combinational therapeutic strategies with established protocols for the prevention and/or treatment of human cancers. Examples of phytochemicals with demonstrated bioactivity against telomerase include (a) isoprenoids – such as perillyl alcohol, genistein, and fisetin, (b) polyphenols – such as curcumin, resveratrol, and EGCG, (c) indole-3-carbinol, and (d) sulforaphane (Table 1).

Among the isoprenoids, perillyl alcohol, a small, lipophilic product of the plant mevalonate biosynthetic pathway [306], [307], [308], suppresses the growth of tumor cells in culture as well as in rodent models [309], [310], [311], [312], [313]. Perillyl alcohol acts on protein translation through modulation of mTOR signaling in cultured prostate cancer cells [314] and in mice with intracranial gliomas [310]. Mechanistically, perillyl alcohol has been shown to disrupt complex formation between mTOR and hTERT, causing p70 S6 kinase (S6K), heat shock protein 90 (Hsp90), and hTERT to dissociate from the regulatory-associated protein of mTOR (RAPTOR) [293], [294].

Genistein – another isoprenoid found in soy and fava beans – has independently been shown to down-regulate telomerase activity and attenuate 4E-BP1 phosphorylation, an indicator of mTOR signaling [314], [315], [316]. Genistein down-regulates hTERT levels by the inhibiting hTERT gene promoter activity in human prostate cancer cells [315]. This study also showed that exposure to genistein inhibited expression of the c-Myc, possibly attenuating its transcriptional activation of the hTERT promoter; however, the inhibitory effects of genistein on hTERT promoter activity likely require the disruption of one or more factors in addition to c-Myc. One contrasting study showed that genistein stimulated hTERT expression in reproductive cancer cells by enhancing STAT3 activity [317], however, the biological context of this observation is not well understood. Consistent with the transcriptional inhibitory effects of genistein, treatment of lung cancer cells with the ethyl acetate fraction of ginger extracts concurrently down regulated hTERT and c-Myc expression, which resulted in a loss of telomerase activity [318]. Genistein also has a strong posttranslational effect on telomerase compartmentalization in prostate cancer cells where it caused down-regulation of the Akt dependent phosphorylation of hTERT and thereby inhibited hTERT translocation into the nucleus [315]. Another structurally similar phytochemical, fisetin, decreases phosphorylation of multiple proteins within the Akt and mTOR pathway, including PI3K, mTOR, S6K and 4E-BP1 [319], [320]. Furthermore, fisetin has shown antitumor efficacy in Lewis mouse lung tumor models [321].

Among polyphenolic compounds, curcumin – a component of the spice turmeric, has also been shown to inhibit telomerase directly and indirectly in human brain, breast, cervical, and leukemic cell lines [322], [323], [324], [325]. This phytochemical reduces mTOR signaling by inhibiting phosphorylation of S6K and 4E-BP1, and disrupts the mTOR–RAPTOR interaction [326], [327], [328]. Like other phytochemicals, curcumin has a range of pleiotropic effects and has been reported to modulate the expression and/or activity of a range of pro- and anti-apoptotic proteins as well as telomerase regulatory factors such as c-Myc and the p53 family [324]. In addition to its effects on mTOR, curcumin modulates additional cellular pathways, including JAK-STAT, NF-κB, and MAPK, that impact telomerase regulation and cancer cell growth [328], [329]. In breast cancer cells, curcumin down-regulation of the levels of the NFkB transcription factor has been proposed to attenuate hTERT expression [330]. In lung cancer cells, curcumin stimulates the level of reactive oxygen species, which triggers the proteasome degradation of the Sp1 transcription factor leading to the loss of hTERT gene expression [331]. Curcumin exposure was also shown to stimulate the cytoplasmic retention of hTERT protein by causing dissociation of hTERT from its chaperone p23 [325]. Resveratrol, another natural phenol produced in many plants including grapes, has been found to decrease hTERT protein levels and inhibit telomerase activity [332], [333]. The combination of curcumin and resveratrol reduced cancer incidence in PTEN knockout mice [334]. EGCG is similar to curcumin in that it was found to modulate multiple oncogenic signaling pathways such as JAK/STAT, MAPK, and PI3K/Akt/mTOR [335]. EGCG down-regulated telomerase activity in multiple carcinoma cell lines and in mouse xenograft tumors [336], [337], [338], [339].

Other phytochemicals with demonstrated telomerase regulatory properties include indole-3-carbinol, a naturally occurring hydrolysis product of glucobrassicin, and the dietary isothiocyanate, sulforaphane, both from cruciferous vegetables such as broccoli and Brussels sprouts. Treatment of cultured breast cancer cells with indole-3-carbinol induced cell cycle arrest and disrupted combined estrogen receptor-alpha and Sp1-driven transcription of the hTERT gene [340]. Chromatin immunoprecipitation analysis of the endogenous hTERT promoter showed that indole-3-carbinol inhibited binding of both estrogen receptor-alpha and Sp1 to a composite estrogen response-Sp1 element in the hTERT promoter [340]. Similar to curcumin, exposure to sulforaphane elevated intracellular reactive oxygen species in hepatocellular carcinoma cells and this process was functionally linked to the inhibition of hTERT gene expression [341]. In hepatocellular carcinoma cells and in breast cancer cells, sulforaphane suppressed Akt kinase activity, which resulted in the loss of hTERT phosphorylation [341].

Candidate telomerase inhibitory natural products are not restricted to phytochemicals. Axinelloside A, a sulphated lipopolysaccharide isolated from the marine sponge *Axinella infundibula*, is found to inhibit telomerase [342]. However, no further studies in relation to telomerase have been reported using this highly complex compound. Telomestatin is a natural macrocyclic pentaoxazole isolated from *Streptomyces anulatus* that inhibits telomerase activity and causes telomere shortening and apoptosis in a range of cancer cell lines *in vitro* as well as leukemia xenografts. Telomestatin also augments apoptosis induced by various chemotherapeutic agents [343], [344], [345], [346]. However, its purification is inefficient and total synthesis is highly complex [347], [348].

## 9 Therapeutic targeting of telomeres

Another plausible approach to the inhibition of telomere maintenance is to directly target telomeres, or to manipulate the shelterin proteins that provide telomere secondary structure and telomerase access (reviewed in [3]). One potential target that has attracted considerable interest is tankyrase 1, a poly(ADP-ribose) polymerase (PARP) family protein that modifies the shelterin protein TRF1 [349], [350]. By modifying TRF1, tankyrase 1 causes displacement of TRF1 from the telomere, thereby promoting telomere unfolding and enabling telomerase to access and lengthen the telomere. General PARP inhibitors have been shown to impede telomere length maintenance and augment the activity of the telomerase inhibitor MTS-312 in experimental systems [349]. Compounds that specifically inhibit the PARP activity of tankyrase 1 are now available for experimental purposes and provide a starting point for the development of tankyrase inhibitors for advancement to clinical trials [351]. Since tankyrase 1 is known to modulate the activity of pathways that that contribute to cancer cell proliferation independently of telomerase (*e.g.*, the WNT/β-catenin pathway), this area of investigation holds considerable promise.

There is a body of evidence that suggests that the 3′ G-rich single stranded overhang of the telomere forms a G-quadruplex structure that is not accessible to telomerase [352], [353]. G-quadruplex stabilizers therefore present a possible alternate means of manipulating telomere maintenance in cancer cells. Molecules that stabilize G-quadruplexes and have been tested for telomere effects include porphyrins (TMPyP4), perylenes (PIPER), acridine derivatives (BRACO19, RHPS4), quinoline-substituted triazines and natural products, such as telomestatin [354], [355], [356], [357], [358]. At non-toxic concentrations, these compounds diminished telomerase enzyme activity, induced telomere shortening and arrested tumor cell proliferation after a lag period [358], [359]. Decreased tumor growth rates were also demonstrated in mice xenografted with leukemia cells [343], [344], [360]. G-quadruplex stabilizing agents may also facilitate the action of standard chemotherapeutic agents and molecular targeted treatments [356], [359]. For instance, telomestatin was shown to enhance the sensitivity of acute myeloid leukemia cells to danunorubicin and cytosine-arabinoside, and was effectively combined with the tyrosine kinase inhibitor imatinib to kill chronic myeloid leukemia progenitor cells [247], [344], [361]. These results clearly illustrate the potential of G-quadruplex stabilizing agents for the treatment of malignancy.

## 10 Other potential therapeutic targets

Because of a lack of definitive molecular criteria, there is currently no consensus regarding properties that contribute to efficient senescence induction. Conceivably, any compound or agent that causes DNA damage or otherwise alters replication may cause senescence at doses that are not cytotoxic. Additionally, agents that stimulate differentiation pathways (such as retinoic acid [362]), may induce senescence in cancer cells that are not able to respond appropriately; this phenomenon which so far has received little attention, can potentially be the basis of truly non-toxic alternative therapies. The therapeutic utility of specific compounds will depend on standard factors such as whether activation is required, uptake and retention, as well as detoxification and repair capacities of the target cells *versus* those of normal cells providing vital functions. Beyond these considerations, however, the specific genetic and epigenetic traits of prospective target cells are likely to have large influences on senescence susceptibility and stability. For example, while conventional genotoxic chemotherapy is often effective at inducing senescence when used at maximally tolerated doses, tumor resistance and recurrences remain significant clinical problems, particularly in patients whose tumors retain intact p53 responses [363], [364]. When treated with genotoxins, wild-type p53 tumors show induction of p21 and SA-βgal, but not p16, and tumor growth often recurs after treatment [364]. Tumor recurrence might be significantly impeded in these cases through the concurrent administration of CDK inhibitors that circumvent defects in the p16/pRB pathway, causing the activation of RB-family dependent heterochromatin formation at E2F-dependent promoters, and stabilization of the senescent phenotype in target cells. Similarly, telomere and telomerase targeting therapies might be improved by the inclusion of agents that induce oxidative stress to discourage the selective outgrowth of ALT+ cells [240].

## 11 Opportunities for senescence drug discovery

In terms of drug discovery, components of the various senescence pathways represent potential targets. To determine whether or not accelerated senescence is a desirable clinical outcome of cancer therapy, it will be necessary to develop efficient and specific approaches to modulate relevant pathways in order to activate the response in a predictable fashion. Thus, ‘proof of concept’ and target validation are necessary before senescence will be seriously considered as an area for widespread commercial drug discovery. Progress in this area has been made through the application of cell-based screening approaches [365]. Cell-based assays offer potential to identify partially validated hits with improved lead-like qualities at the earliest stages of discovery and offer a number of critical advantages over conventional biochemical screening assays. Purification of the target protein in a functional conformation is not required. Instead, the key requirement is a measurable marker of endogenous target inhibition such as a change in reporter activity, protein phosphorylation, or cell morphology. Importantly, cell-based assays may also discriminate between different drug effects, such as antagonism *versus* agonism, and identify hits that interact with different target conformations that may be present in a physiological setting. The characteristic morphological changes associated with cell senescence including cell enlargement and SA-βGal activity are suitable for cell based screening approaches using high content automated imaging. The validity of this approach has recently been demonstrated in both normal and cancer cell-based screening strategies using siRNA or small molecule libraries to identify compounds that modulate senescence and targets for further validation [366], [367]. Interestingly, in these studies, senescence occurs in a matter of days post treatment, an advantage over the longer lag time generally associated with telomerase inhibition.

Thus senescence is already beginning to show great promise as an endpoint for drug targeting. Cell-based senescence models and assays have contributed considerably to advancing new approaches to drug development. Importantly, a number of agents already used in the management of human cancers also show associations with senescence phenotypes, opening up opportunities for drug re-positioning [80], [365]. However, the key questions as to which patient group will benefit from such agents and clinical trial design are still open to debate and likely to revolve around the specific target in question.

## 12 Cross-validation

Given the heterogeneity present in most cancers, complete arrest/eradication of the various subpopulations of cells in any given cancer will likely require simultaneous targeting of several molecules and mechanisms that contribute to the malignant phenotype. It is therefore important to anticipate complementary as well as antagonistic effects on different cancer hallmarks that might be achieved using agents directed against specific targets. Accordingly, a literature search has been conducted by a “cross-validation” team to identify potential consequences of the use of selected agents targeting protein complexes involved in the maintenance of replicative immortality on other hallmarks of cancer. The results of this survey are listed in Table 2. The table indicates that not all interactions are favorable; some agents efficacious against replicative immortality may exacerbate other hallmarks (*e.g.*, genetic instability), and their utility may be context-dependent.

## 13 Conclusions

Developing optimized and truly holistic cancer prevention and treatment regimens will likely incorporate strategies that target replicative immortality. The chief advantage to be gained by the use of these senescence-inducing regimens is elimination of tumor repopulating ability with reduced collateral damage compared to conventional cytotoxic regimens. There are certain questions and risks associated with this strategy that must be addressed before its wholesale adoption in clinical settings. In the case of telomere and telomerase based strategies, replicative senescence may occur more readily in cancer cells bearing short telomeres than in vital normal cells with long telomeres, but telomere lengths in cancer cells may still be long enough to permit sufficient population doublings for invasion and metastases to occur. Moreover, telomere dysfunction promotes the development of chromosomal instability, which in turn can generate mutations that enable cells to become drug resistant and/or activate ALT mechanisms for telomere maintenance and/or become more malignant. High priority should be given to further research into the determinants of senescence stability, as the implications of delayed cell cycle re-entry, permanent cytostasis, or eventual clearance may be profoundly different. Lower doses of genotoxic drugs needed to induce senescence may reduce collateral damage, but allow establishment of dormancy by resistant cells. Conversely, since it is almost impossible to kill all the cells in a tumor even at the highest tolerated doses of chemotherapy, addition of a complementary agent that induces or enhances stable senescence in the cancer cells that manage to retain viability might additively or synergistically increase therapeutic efficacy. The microenvironmental and systemic effects of senescent cells also need further clarification. While senescent cells have been shown to secrete proteins that aid in their own clearance and tissue repair, persistent senescent cells can disrupt niches, causing the depletion of healthy stem cells, and can promote aging and cancerous phenotypes in surrounding tissues. Given that some senescence-associated secretory phenotypes can be pro-tumorigenic, is senescence still a good objective? Can a proper immune response be counted on to clear senescent cells? Ultimately, given the hypermutability and heterogeneity of most human cancers, it is often unlikely that targeting any single gene product or pathway will provide lasting remission. However, developing agents with good mechanistic understanding, that effectively disrupt replicative immortality, may well add to the arsenal available for combinatorial approaches.

## Conflict of interest

Dr. Keith has collaborative agreements with AstraZeneca and Geron Corporations, and is founder and director of Senectus Therapeutics.

## Figures and Tables

**Table 1:** Therapeutic targeting by select phytochemicals.

Phytochemical	Targets	Current state of evidence
Perillyl alcohol	mTOR [292], [293], [294], [295], [296], [310], [314] Telomerase [292], [293], [294], [295], [296] Ras-Erk [309], [310]	Cell lines [292], [293], [294], [295], [296], [310], [311], [314], [368] Animal models [307], [308], [309], [310], [311] Clinical trials [369], [370], [371], [372]
Curcumin	mTOR [326], [327], [373], [374] Telomerase [322], [323], [324], [325], [375] Akt [326], [373] Hsp90 [325] NF-κB [328], [375] JAK/STAT [328], [376] MAPK [328], [329]	Cell lines [322], [323], [324], [325], [326], [327], [373], [375], [376] Animal models [334], [373], [374], [375] Clinical trials [377], [378], [379], [380]
Resveratrol	mTOR [334], [381] Telomerase [332], [333] Akt [334], [382] MAPK [383], [384]	Cell lines [332], [333] Animal models [334], [381], [384] Clinical trials [385], [386], [387]
EGCG	mTOR [335], [388] Telomerase[336], [337], [338], [339] PI3K[335], [388] NF-κB [335], [389] JAK/STAT [335], [390] MAPK [335], [391]	Cell lines [336], [337], [338], [339], [389], [390], [392], [393] Animal models [337], [338], [394] Clinical trials [395], [396], [397]
Genistein	mTOR [314], [398] Telomerase [315], [316] Akt [314]	Cell lines [314], [315], [316] Clinical trials [399]
Fisetin	mTOR [319], [320] PI3K/Akt [319], [320]	Cell lines [319], [320] Animal models [321]

**Table 2:** Cross-validation of selected targets and agents.

Targets for replicative immortality	Telomerase (inhibit)	hTERT (inhibit)	mTOR (inhibit)	CDK4/6 (inhibit)	CDK1/2/5/9 (inhibit)	Akt (inhibit)	PI3K (inhibit)
*Other cancer hallmarks*^a^							
Genomic instability	+/− [400], [401], [402]^c^	+/− [400], [401], [402]	0	− [403]	+ [404], [405]	+ [406], [407], [408]	+ [409]
Sustained proliferative signaling	+ [410]	+ [411], [412], [413]	+ [414], [415], [416]	+ [417], [418]	+ [419], [420], [421]	+ [415], [422], [423]	+ [424], [425]
Tumor promoting inflammation	+ [410]	+ [410]	+ [426], [427]	+ [428]	+ [429], [430]	+ [431], [432]	+ [433], [434]
Evasion of anti-growth signaling	+ [258], [276], [435]	+ [258], [276], [435]	+/− [436], [437]	+ [438], [439]	+ [440], [441], [442]	+ [443], [444]	+ [445], [446]
Resistance to apoptosis	+ [447]	+ [448]	+ [449]	+ [450]	+ [451]	+ [452]	+ [452]
Dysregulated metabolism	+ [453]	0	+ [454]	+ [455]	+ [456]	+ [457], [458]	+ [459], [460]
Immune system evasion	0	0	+/− [461]	0	0	+ [462]	+/− [463], [464]
Angiogenesis	+ [465]	+ [465]	+ [466], [467]	+ [468], [469]	+ [470], [471]	+ [466]	+ [466]
Tissue invasion and metastasis	+ [472], [473]	+ [472], [473]	+ [474], [475]	+/− [476], [477]	+ [478], [479]	+ [480]	+ [475]
Tumor microenvironment	+ [481]	+ [482]	+ [483]	+ [484]	− [485], [486]	+ [487], [488]	+ [488], [489]

Approaches	GRN163L (imetelstat)	Genistein	Perillyl alcohol	PD 0332991 (palbociclib)	SCH 727965 (dinacicilib)	Curcumin	EGCG (epigallocatechin-3-gallate)
*Other cancer hallmarks*^b^							
Genomic instability	+/− [490], [491]	+/− [492], [493]	+ [494]	0	0	+ [495], [496]	+/− [497], [498]
Sustained proliferative signaling	0	+/− [499], [500]	+ [501], [309]	+ [502]	+ [503]	+ [504], [505]	+ [506], [507]
Tumor promoting inflammation	0	+/− [508], [509], [510]	+ [309], [511]	0	0	+ [512], [513], [514]	+ [515], [516]
Evasion of anti-growth signaling	+ [258]	+ [517], [518], [519]	+ [520]	+ [502], [521]	+ [522]	+ [523], [524]	+ [525], [526]
Resistance to apoptosis	+ [276]	+ [527], [528]	+ [529], [530]	+ [502], [531]	+ [522], [532]	+ [533], [534]	+ [535], [536]
Dysregulated metabolism	0	+ [537]	+ [310]	0	0	+ [538]	+ [539], [540]
Immune system evasion	0	+ [541]	0	0	0	+ [542], [543]	+/− [544], [545], [546]
Angiogenesis	0	+ [547], [548], [549]	+ [550]	0	0	+ [551]	+ [552], [553]
Tissue invasion and metastasis	0	+/− [554], [555], [556]	+ [310]	− [476]	+ [503]	+ [557], [558]	+ [559], [560], [561]
Tumor microenvironment	+ [562]	+ [563]	+ [550]	0	+ [564]	+ [565], [566]	+ [567]

a Potential consequences of targeting indicated protein complexes involved in the maintenance of replicative immortality on other hallmarks of cancer: +, inhibition of indicated target has beneficial consequences for hallmark; −, inhibition of indicated target exacerbates hallmark; +/−, inhibition of indicated target has both positive and negative effects on hallmark; 0, no published evidence of any effect on hallmark.

b Potential consequences of the use of selected agents targeting pathways involved in the maintenance of replicative immortality on other hallmarks of cancer: +, indicated agent has beneficial consequences for hallmark; −, indicated agent exacerbates hallmark; +/−, indicated agent has both positive and negative effects on hallmark; 0, no published evidence of any effect of indicated agent on hallmark.

c Numbers in brackets refer to references containing evidence for potential consequences listed.
